# Quality of Life in Female Patients Following Ileal Neobladder and Ileal Conduit: Where Are We?

**DOI:** 10.3390/jcm10143042

**Published:** 2021-07-08

**Authors:** Salvatore Siracusano, Agustina Zaka, Federico Romantini, Antonio Benito Porcaro, Carlo Vicentini, Cristina Lonardi

**Affiliations:** 1 Department of Life, Health and Environmental Sciences, University of L’Aquila, 67100 L’Aquila, Italy; federomantini@gmail.com (F.R.); carlo.vicentini@univaq.it (C.V.); 2 Department of Urology, University of Verona, 37129 Verona, Italy; zakaagustina@gmail.com (A.Z.); drporcaro@yahoo.com (A.B.P.); 3 Department of Human Science, University of Verona, 37129 Verona, Italy; cristina.lonardi@univr.it

**Keywords:** female, muscle-invasive bladder cancer, quality of life

## Abstract

Women undergoing a radical cystectomy (RC) followed by a urinary diversion (UD) for bladder cancer (BC), experience a substantial reduction in health-related quality of life (HRQOL). At present, studies comparing long-term QOL outcomes for different UD methods in female patients are lacking. We summarize the current state of the QoL assessment in female patients after an RC.

## 1. Introduction

Bladder cancer (BC) is one of the most frequent types of cancer in males and females, and it is the sixth in the United States of America, counting 81,190 new estimated cases in 2018 [1,2].

Radical cystectomy (RC) using a lymph node dissection with an ileal conduit (IC) or an orthotopic neobladder (ONB) urinary diversion, is the standard treatment, recommended by the European Association of Urology (EAU) guidelines, for localized muscle-invasive bladder cancer [3] Furthermore, RC is also a recommended treatment for non-muscle-invasive bladder cancer (NMIBC) when non-responsive to standard treatments.

RC often deeply affects patients’ lives, bringing many changes in their daily activities, and physical and mental health. RC is a traumatic and invasive event for the patients, with repercussions on their daily, social, working, and sex life. Such changes, and subsequently the Quality of Life (QoL) of patients, are commonly studied, before and after the operation, via validated questionnaires.

To date, studies focusing on functional outcomes, sexual function, and health-related QoL for both genders are lacking, especially regarding female patients, who have statistically significantly worse overall survival, recurrence-free survival, and cancer-specific survival rates [4].

The purpose of this article is to review and summarize recent data on QoL, as well as oncologic and functional outcomes, in women undergoing radical cystectomy with urinary diversion.

## 2. Measurement of Quality of Life

Questionnaires are the most commonly used tools to assess QoL in patients. There are many different questionnaires, ranging from cancer-specific to more general ones.

In this regard, patient-reported outcome measures (PROMs), such as questionnaires used to assess QoL in cystectomized patients, are useful and essential tools used by clinicians to understand patient-focused outcomes.

Frequently, the items assessed by such instruments regard physical, psychosocial, health- and disease-specific aspects of a patient’s life; a score is assigned to all the aspects, and a final score on the person’s current life satisfaction is obtained.

Recently, bladder cancer-specific questionnaires have been developed, and urologists worldwide broadly use them, during the follow-up of patients after RC [5].

The RAND Medical Outcomes Study 36-Item Health Survey (SF-36) is a survey, which includes 36 questions about physical function, mental health, vitality, pain, and social life, and it is the most widely used tool to study the general QoL of patients [6]. Although the SF-36 does not contain specific items related to cancer, it has been used to assess the general quality of life in patients with bladder cancer undergoing cystectomy. The SF-36 is among the most validated and responsive general quality-of-life instruments, yet it lacks the specificity to gauge cancer-specific issues.

Nowadays, many new questionnaires, regarding patients affected by BC, have been validated, and they are categorized into two large groups: Instruments that are treatment-neutral and treatment-specific, in relation to the urinary diversion [7].

The Functional Assessment of Cancer Therapy (FACT-G), is a cancer-patients-specific questionnaire, commonly used by clinicians to address cancer-related issues, such as pain, vomiting, nausea, and sleep quality [8]. The FACT-G comprises of 27 items and four subscales: Physical Well-Being (PWB), Social/Family Well-Being (SWB), Emotional Well-Being (EWB), and Functional Well-Being (FWB). The FACT score rates the health-related quality of life in patients treated for cancer. The score sums up to a total, ranging from zero to 108 points, where a higher score indicates better quality of life.

The European Organization for Research and Treatment of Cancer Quality of Life Core Questionnaire-Bladder Cancer Muscle-Invasive 30 (EORTC-QLQ-BLM 30), is a 30-question questionnaire, specifically developed for patients affected by muscle-invasive bladder cancer [9]. It contains multi-item scales that assess urinary symptoms, urostomy problems, future perspectives, abdominal bloating, body image, sexual function, and catheter use problems. It is intended to be administered in addition to the EORTC QLQ-C30 items, which contains items within 15 domains pertaining to physical, role, emotional, cognitive, and social functioning, as well as items related to fatigue, nausea, vomiting, pain, dyspnea, insomnia, appetite loss, constipation, diarrhea, and financial difficulties.

The Functional Assessment of Cancer Therapy—Vanderbilt Cystectomy Index (FACT-VCI), is a 17 items questionnaire regarding urinary and sexual symptoms in patients with either ileal conduit or orthotopic neobladder [10]. It is made of 17 questions, each of which is scored with a 5 points scale, from 0 for ‘not at all’ to 4 for ‘very much’, with higher scores indicating a better quality of life. The scores are then added into a single score without domains. The questions specifically relate to urinary, bowel, and sexual function.

The Functional Assessment of Cancer Therapy—Bladder Cancer (FACT-BI), consists of 27 questions, which cover similar fields of the FACT-VCI, but has two more questions for patients with a stoma [11]. It evaluates the physical, social, family, emotional and functional well-being, as well as a condition-specific component that assesses issues related to bladder cancer, such as urinary, bowel, and sexual function. The questionnaire, however, lacks a bother subscale, and it cannot discriminate which symptoms are the most bothersome.

The University of Michigans’s Bladder Cancer Index (BCI) is a disease-specific index comprising of 34 items regarding three domains: Urinary and sexual function, as well as bowel function. Item responses are given on a scale from 0 to 100, with higher scores indicating better function [12]. The bother subscale offers the ability to measure the impact of the symptoms on the everyday-life of the patient.

For sexual life assessment, so far, literature is mainly focused on men, showing a lack of female-targeted questionnaires. In this field, the most widely used instrument is the Female Sexual Function Index (FSFI) [13]. It is made up of 19 questions, each of which can have a score from 0 to 5, and all of the items take into consideration the sexual satisfaction of the patient during the last four weeks.

Recently, new PROMs have been developed to further improve the measurement of the QoL. One of such new models is the Ileal Orthotopic Neobladder PRO questionnaire (IONB-PRO), made of two sections: One on the QoL and a second section on the capability of the patient to manage the IONB. To evaluate the QoL, it uses three different versions: A basic 23-item QoL version; a short-form 12-item QoL scale, and a short-form 15-item Rasch QoL scale [14]. The domains of the questionnaire are symptoms, neobladder self-management, activities of daily living, emotional issues, social issues, sleep fatigue, while the specific items are about incontinence, urinary retention, daily life organization, irritability, coping, fear, and about other social and psychological aspects of the patient’s life.

### 2.1. Quality of Life in Cystectomized Female Patients

Women undergoing a cystectomy have a shorter survival span than men. This aspect could be due to a different gene expression of urothelial cancer in women compared to men, since it has now been reported that bladder cancer in women often shows a squamous or sarcomatoid component [15]. Other authors have also hypothesized that estrogen receptors subtype-B are often responsible for a highly aggressive urothelial tumor [16]. All these elements have contributed to have a scarce number of long-term survivors among women. Consequently, it is difficult to evaluate the QoL in female subjects undergoing cystectomy.

In this context, the current literature does not appear unanimous for both sexes in defining the IC as a better derivation than the ONB, in terms of quality of life. There are papers that favor the IC as a better derivation than ONB, while others favor ONB, and still others that have shown similar results between the two [17]. Three retrospective studies favored the IC diversion over ONB by using the FACT-VCI scores showing that patients with an IC diversion had a total score that averaged higher than those with ONB, after 12 months [18,19,20]. Erber et al. [21] have demonstrated better results in global health stats and the QoL of ONB than IC, after long-term follow-up, and finally, six studies, presented similar results between the two urinary diversions (Table 1).

However, sexual life in women after a cystectomy represents a major concern, for physicians and patients. In fact, while in men, a nerve-sparing cystectomy can be effective for the recovery of sexual activity, in women, this aspect is not clearly verified. The neurovascular bundles, located on the lateral walls of the vagina, are usually removed or damaged during surgery. Moreover, a significant devascularization of the clitoris can occur during the removal of the distal urethra. In this way, the anterior vaginal wall is usually removed en-block, resulting in a narrowed vagina. As reported by others, the most common complaint after an RC is represented by a reduced ability to achieve an orgasm, in 45% of patients, by decreased lubrication in 41% of the cases, with a significant decrease of sexual desire and an incidence of dyspareunia in 37% and 22% of cases, respectively [30]. In this context, several authors have attempted to preserve sexual function after a cystectomy in women by proposing nerve preserving surgery through the sparing of the innervations of the clitoris and distal urethra in selected cases, to maintain vaginal lubrication, but to date, there is no relative information on the quality of life and sexuality of these patients who have undergone this kind of surgery. These elements could explain QoL issues in female patients.

In fact, to date, the majority of the manuscripts on this topic are about general QoL studies regarding both female and male patients, but rarely on the female population only (Table 2).

In 2013, a multicenter study on 37 female patients who had undergone radical cystectomy and urinary diversion, either IC, cutaneous ureterostomy (CUS) or ONB, showed that, after a follow-up period of 36 months, the QoL of the patients who had undergone the cutaneous ureterostomy was lower, when compared to the IC or ONB group [31]. The QoL was assessed using the EORTC, QLQ-BLM30, and QLQ-C30 questionnaires, and all the patients were disease-free at the follow-up. The most significant differences were found in the physical and emotional perception of their body image. In the EORTC QLQ C-30, the CUS subgroup scored higher for appetite loss and fatigue, with statistically significant differences. In the QLQ BLM-30, the CUS subgroup scored worse for physical and emotional well-being.

In 2014, Messer et al. in a large, multicenter, observational, cohort study using retrospectively collected data on 4296 cystectomized patients treated with RC, including 890 women, showed an increased risk for disease recurrence and cancer-specific mortality in the female population [32].

In 2018, a study on 145 patients undergoing radical cystectomy with IC urinary diversion, took into consideration both males and females, providing a comparison of the two groups. QoL was assessed by EORTC QLQ-C30 and EORTC BLM-30. The female patients were 33, and they presented significantly greater problems than men in cognitive functioning and in future perspective [33].

Another study, by the same author, explored the differences in female patients following two different urinary diversions, regarding IC vs. ONB. Seventy-three female bladder cancer patients were studied after undergoing RC, using the EORTC QLQ-C30 and EORTC QLQ-BLM30 questionnaires. The two groups had no statistically significant differences in pathological stage, grade, chemo- or radiotherapy, even though the ONB group was significantly younger than the IC group. The study showed that, based on long-term follow-up, there were no significant differences in the QoL of the two groups, except for financial difficulties, which appeared to affect the ONB patients more than the IC patients. For the authors, this difference, could be, at least in part, explained by the age difference, with the oldest group being less financially fragile [34].

A study by Singer et al. analyzed the QoL of 823 patients, 235 of which were female. The study found that 70% of the females had conduit, 15% had an ONB or a pouch, while 49% of men had an ONB, 50% had a conduit, and only 1% had a pouch. Men were nine times more likely to receive ONB than women. All 823 patients reported a decreased QoL when compared to the general population, but women scored lower values, both in the patient’s category and the general population comparison group. The main differences were found in fatigue, physical functioning, and role functioning [35].

### 2.2. Quality of Life in Female vs. Male

To current date, there are only a few studies that try to review and summarize recent data on gender differences in oncologic and functional outcomes in women, comparing them to male patients, undergoing radical cystectomy with urinary diversion. Contemporary studies have highlighted a potential disparity in outcomes between men and women.

Moreover, larger studies that directly examine the quality of life, as well as the functional outcomes, examining gender-specific urinary and sexual function, are lacking.

A review by Sadighian et al. concluded that gender negatively affects oncologic outcome after treatment for bladder cancer, with women being the weaker factor of the equation. In this setting, varying socioeconomic circumstances and biological differences in cancer initiation, as well as the response to therapy, seem to be responsible for overall poorer quality of life in bladder cancer female patients, when compared to their male counterpart [36].

The few studies addressing female sexual function before and after radical cystectomy have used the Female Sexual Function Index (FSFI), which is one of the few validated questionnaires currently available, consisting of 19 questions concerning: Desire, arousal, lubrication, orgasm, satisfaction, and pain following vaginal penetration. Gontero et al. focused on the impact of radical cystectomy on the sexual life of both female and male patients, reviewing case series of female QoL and the impact of surgery on their sexual life [36]. The conclusion of this review is that sexual dysfunction, following radical pelvic urological surgery adversely affects the overall QoL, of both men and women, with the substantial difference that men have been the main focus of recent papers and research efforts, mainly focused on erectile dysfunction, while little is known about the extent of female sexual dysfunction after radical pelvic surgery.

Recently, the paper by Siracusano and co-workers focusing on the differences between male and female bladder cancer patients, compared 112 men vs. 33 women, and although the two groups were comparable in terms of clinical and demographical variables, the differences in the overall QoL were significantly worse for women than for men, but when specifically enquired about their sexual life, men had more problems in sexual functioning, and in general sexual satisfaction, than women [33].

## 3. Discussion

Current literature underlines the importance and significance of QoL assessing of bladder cancer patients undergoing RC with urinary diversion. Historically, science and medicine in general, have been focused solely on the male patient, and only in recent years, we have lived a shift towards a more gender-neutral environment. Anatomical and psychological differences between men and women should always be taken into consideration before and after major surgery, more so after RC, which deeply impacts the everyday life and sexual sphere of a patient. Unfortunately, today we only have a handful of studies that have tried to assess the QoL of females after such intervention, or tried to compare it to its male counterpart. A review of such studies suffers from the disparity of the tools used, and some studies enquired the patients after 12 months, while others after 6 months, making the interpretation of data difficult.

In general, it has been shown that women have a lower survival rate after cystectomy than men, and at the same time, a worse QoL. However, this aspect is strongly undermined by the absence of questionnaires on the QoL after a cystectomy, specifically dedicated to the female sex. In fact, modern questionnaires have been validated mainly on a male population. This bias is certainly the cause of incomplete information on the QoL in women, when compared to men. Even regarding sexuality, female problems are often underestimated compared to the erectile dysfunction of males. The surgical optimization carried out until now, with the execution of a nerve and vaginal sparing surgery, does not allow us to express any opinion regarding the quality of life of these patients.

Women seem to have a lower QoL, with major concerns arising from their impacted body image perception and cognitive functions [37]. Their sexual life and quality seem to suffer less from the RC, and this is probably due to erectile dysfunction in men is more common than pain, lubrication, and difficulties of penetration in women. The factors that account for sexual dysfunction in women who have undergone RC and urinary diversion are surgical factors, including technique and choice of diversion, psychological burden of cancer diagnosis, oncological outcomes, hormonal status, relationship with current partner, post-operative continence, and concomitant comorbidities.

In this context, it should also be stressed that a factor that can affect the QoL in these patients is hypercontinence, and in this occurrence many of these patients need self-catheterization. In several cases, to avoid hypercontinence, the surgeon tries to reconstruct the posterior bladder support, preserving the vagina and affixing the omentum to the perineum, under the neobladder. Currently, there are few experiences in this regard, and therefore, this aspect remains a reason for further studies [38].

It is evident that treating female patients with BC is a challenging task for urologists. This is not only because of the possible mismatch between surgical, oncological, and functional outcomes, but also because the female gender represents a risk factor for poor surgical and oncological results following RC, thus, directly affecting the patient’s QoL after the RC. The urological literature stresses the importance of QoL in patients undergoing RC and urinary diversion. Unfortunately, only a few studies have compared the QoL between female and male patients, who present different types of post-operative complications and impact on daily and personal life, and even fewer papers focus solely on female patients.

## 4. Conclusions

Actually, QoL in females following an RC remains a challenging issue, and prospective and controlled studies focusing on female QoL are specifically required.

## Figures and Tables

**Table 1:** Characteristics of included studies.

Study	Year	Study Type	No (IC/NB)	Scale Used	Conclusion on QoL
Anderson [18]	2012	Retrospective	172 (71/101)	FACT-VCI	IC better than ONB
Erber [21]	2012	Retrospective	58 (24/34)	EORTC QLQ-C30 EORTC QLQ-BLM30	ONB better than IC
Metcalfe [22]	2013	Retrospective	84 (53/31)	FACT-VCI	No difference
Singh [23]	2014	Retrospective	164 (80/84)	EORTC QLQ-C30	ONB better than IC
Large [24]	2014	Retrospective	43 (27/16)	FACT-VCI	No difference
Goldberg [20]	2015	Retrospective	95 (49/46)	BCI	IC better than ONB
Huang [25]	2015	Retrospective	117 (78/39)	BCI BIS	No difference
Kretschmer [26]	2016	Retrospective	100 (50/50)	EORTC QLQ-C30	No difference
Cerruto [27]	2016	Retrospective	319 (148/171)	EORTC QLQ-C30 EORTC QLQ-BLM30	No difference
Gellhaus [28]	2016	Retrospective	92 (44/48)	BCI	IC better than ONB
Zahran [29]	2017	Retrospective	145 (61/84)	EORTC QLQ-C30 FACT-B1	No difference

IC: ileal conduit; NB: neobladder; QoL: quality of life; ONB: orthotopic neobladder.

**Table 2:** Studies published on QoL in cystectomized female patients (CUS, cutaneous ureterostomy; ONB, orthotopic neobladder; IC, ileal conduit).

Author	Number of Female Patients	Urinary Diversion	Administered Questionnaire
Gacci M. 2013	37	CUS–ONB–IC	EORTC QLQ-BLM30 QLQ-C30
Siracusano S. 2018	145	IC	EORTC QLQ-C30 EORTC BLM-30
Siracusano S. 2018	73	ONB–IC	EORTC QLQ-C30 EORTC QLQ-BLM30
Singer S. 2012	235	ONB–IC	EORTC QLQ-C30
Gontero P. 2006	56	ONB–IC	FSFI

CUS: cutaneous ureterostomy; ONB: orthotopic neobladder; IC: ileal conduit.

## Data Availability

All data analyzed in this paper was obtained from: https://pubmed.ncbi.nlm.nih.gov/database.
